# Association between serum 25-hidroxyvitamin D concentrations and ultraviolet index in Portuguese older adults: a cross-sectional study

**DOI:** 10.1186/s12877-017-0644-8

**Published:** 2017-10-31

**Authors:** Sofia Cardoso, Alejandro Santos, Rita S. Guerra, Ana S. Sousa, Patrícia Padrão, Pedro Moreira, Cláudia Afonso, Teresa F. Amaral, Nuno Borges

**Affiliations:** 1 Faculdade de Ciências da Nutrição e Alimentação da Universidade do Porto Rua Dr. Roberto Frias, 4200-465 Porto, Portugal; 2 I3S-Instituto de Investigação e Inovação em Saúde, R. Alfredo Allen, 4200-135 Porto, Portugal; 3 UISPA, LAETA-INEGI, Faculdade de Engenharia, Universidade do Porto, Porto, Portugal; 4 EPIUnit, Instituto de Saúde Pública, Universidade do Porto, Rua das Taipas, n° 135, 4050-600 Porto, Portugal; 5 Centro de Investigação em Atividade Física, Saúde e Lazer, Universidade do Porto, R. Dr. Plácido da Costa 91, 4200-450 Porto, Portugal; 6 CINTESIS - Centre for Health Technology and Services Research, Rua Dr. Plácido da Costa, 4200-450 Porto, Portugal

**Keywords:** Vitamin D, Vitamin D deficiency, Older adults, Cutaneous synthesis, 25-hydroxyvitamin D3, Ultraviolet index

## Abstract

**Background:**

The older population is a risk group for hypovitaminosis D. The Ultraviolet Index (UVI) can be an indicator of potential for cutaneous synthesis of vitamin D but physiological and other environmental factors also influence vitamin D synthesis and status. Knowledge about vitamin D status in Portuguese older adults is limited. This study aims to explore the association between Ultraviolet Index and serum 25-hidroxyvitamin D3 [25(OH)D] levels accounting for other potential influential factors.

**Methods:**

A cross-sectional study was conducted between December 2015 and June 2016, in 1497 Portuguese older adults (≥ 65 years) within Nutrition UP 65 project. For each participant, serum 25(OH)D was determined and the mean UVI (mUVI) in the respective residence district was calculated for the previous 30 days. Stepwise linear regression analyses were conducted for the following periods of blood collection: between December and June, December and March and April and June. Standardized regression coefficients (Sβ) and 95% confidence intervals were calculated.

**Results:**

The median 25(OH)D concentration was 35.9 nmol/L. The UVI was independently and positively associated with 25(OH)D in the models for December–June (Sβ = 0.244, 95% CI: 0.198; 0.291, *P* < 0.001) and April–June (Sβ = 0.295, 95% CI: 0.299; 0.362, *P* < 0.001) and independently and negatively associated in December–March period (Sβ = −0.149, 95% CI: -0.211; −0.087, *P* < 0.001).

**Conclusions:**

In this sample with high vitamin D deficiency frequency, the UVI was a predictor of 25(OH)D levels but the direction of the association varied according to the blood collection period. Our results suggest that accounting for the time of year in future research regarding vitamin status and related public health recommendations may be relevant.

**Electronic supplementary material:**

The online version of this article (10.1186/s12877-017-0644-8) contains supplementary material, which is available to authorized users.

## Background

Vitamin D deficiency may be considered a pandemic in Europe and a global public health problem that also entails an economic burden [1, 2]. Older adults, aged 65 years and older are a risk group for vitamin D deficiency [3] and consequences include osteomalacia and increased risk of muscle weakness, falls and fractures [4, 5]. Hypovitaminosis D has also been associated with an increased risk of several morbidities including neuropsychiatric, such as dementia [6], and cardiovascular diseases [7].

Sources of vitamin D include solar ultraviolet B (UVB) radiation, diet and supplements. Sun exposure can be considered the main source [4], but at some latitudes and/or seasons, it is not enough to maintain an adequate status and diet or supplements may have a more important role [3].

The cutaneous synthesis of vitamin D is influenced by environmental factors such as UVB radiation available, which is a function of the solar zenith angle that varies with latitude, season and time of day, as well as individual factors such as age, skin pigmentation and sun exposure behaviours [8]. As latitude and age increase, potential to synthesize vitamin D decreases.

Vitamin D synthesis usually increases from spring to summer and decreases afterwards [4]. This results in a seasonal variation of 25(OH)D serum concentrations, which reaches its nadir in winter/early spring [1, 9]. Researchers have described a “vitamin D winter” referring to the months during which solar UV radiation is not intense enough to allow synthesis [9, 10] and occurs at latitudes above approximately 35°-40 °N [8, 11–13].

The UV index (UVI) is a parameter that describes the level of UV radiation at the Earth’s surface that can cause erythema [14]. Additionally, the UVI can be an indicator of potential for cutaneous synthesis of vitamin D: the higher the UVI the greater the potential [15]. In most studies, sun exposure and UVI are associated with 25(OH)D concentrations [16–19], but investigation in vivo is still complex. Several studies have also focused on the effect of latitude and season on 25(OH)D. UVI provides more information than latitude by itself, because it’s dependent on solar zenith angle and accounts for other variables that affect UV radiation available for synthesis. This study will explore the association of UVI and 25(OH)D concentrations accounting for the effect of other variables such as sociodemographic, health and anthropometric data, which were not included in previous studies that evaluated sun exposure or just the association between UVI and vitamin D status.

Current knowledge about the vitamin D status of elderly in Portugal is limited [20].

In other countries in similar latitudes, such as Spain [21–23] and Italy [24, 25] as well as in Europe, America and Australia more studies have been published in this area [26, 27]. The investigation of the association between UVI and 25(OH)D was not done in older adults specifically [17–19, 28].

Since population ageing is accelerating [29] and given the pleiotropic effects of vitamin D and the possibility in modifying disease risk [9], the study of the determinants of vitamin D status, namely its association with the UVI, could improve knowledge and provide useful information for future research and guidelines that help to face this problem.

The aim of this study was to explore the association between UVI and serum 25-hidroxyvitamin D3 [25(OH)D] levels, considering the effect of other potential influential factors, in Portuguese older adults.

## Methods

### Study design and setting

The present study is a cross-sectional study with a sub-sample of 1497 older adults (≥ 65 years) from the Nutrition UP 65, whose data collection took place between December 2015 and June 2016. The “Nutrition UP 65 Study - Nutritional Strategies Facing an Ageing Demography” is a cross sectional observational study conducted in Portugal, in a sample of 1500 subjects (≥ 65 years old). The main objectives were to reduce the nutrition inequalities and provide knowledge about nutritional status of this population [20]. The complete description of Nutrition UP 65 can be read elsewhere [20].

Three individuals were excluded from the original sample: one subject presented a serum 25(OH)D concentration above the toxicity level of 375.0 nmol/L [9] and two subjects were excluded due to missing data.

All data of this study were obtained within the Nutrition UP 65 except for the UVI and latitude data.

### Sampling and recruiting

A quota sampling approach regarding sex, age, educational level and regional area was implemented to obtain a representative sample of Portuguese older adults. Eligible individuals were Portuguese, with 65 years of age or more. The sample was constituted by community-dwelling and 5% of institutionalized individuals, according to the previously described proportion [29].

The regional areas used were the ones defined in the Nomenclature of Territorial Units for Statistics (NUTS II): Alentejo, Algarve, Azores, Lisbon Metropolitan Area, Center, Madeira, and North. A random and stratified cluster sampling was applied. In each regional area, three or more town councils with more than 250 inhabitants were randomly selected.

Potential participants were informed about the Nutrition UP 65 and were invited to participate.

### Study locations

In total, 15 districts were selected: 13 districts of mainland Portugal which were Aveiro (40.9 °N), Braga (41.5 °N), Coimbra (40.2 °N), Évora (38.7 °N), Faro (37.1 °N), Leiria (39. °N), Lisboa (38.9 °N), Portalegre (39.5 °N), Porto (41.2 °N), Santarém (39.4 °N), Setúbal (38.6 °N), Viana do Castelo (41.7 °N) and Viseu (40.8 °N), in addition to Ponta Delgada from the Azores Archipelago (38.3°N) and Funchal (32.7 °N) from the Madeira Archipelago.

### Ultraviolet index

The UVI forecast was provided by the Portuguese Institute of Sea and Atmosphere (IPMA), for the studied districts and for the period between November 2015 and July 2016. The forecast corresponded to the daily maximum UVI around the solar noon and was obtained by the German Meteorological Service (DWD), whose forecasting has a module structure. The UVI is calculated based on defined values of aerosol optical depth and type and of surface UV albedo. It depends on the solar zenith angle and forecasted ozone columns; it is also adjusted for other variables, such as altitude and cloudiness [30].

### Data collected in Nutrition UP 65 Study

The following information were collected through a structured questionnaire (please see Additional files 1 and 2): cognitive performance; sociodemographic data (sex, age, educational level, professional activity, marital status, residence and household income); lifestyle (current smoking habits, consumption of alcoholic beverages, physical activity and adherence to Mediterranean Diet); skin phenotype; health status and clinical history. Nutritional and vitamin D status were also evaluated. The interviews were carried by previously trained registered nutritionists.

Skin phenotype was self-reported by the participants and classified according to Fitzpatrick (1975) scale that comprises six categories (I-VI) in ascending order of pigmentation [31].

Cognitive performance was assessed by the Portuguese version of the Mini Mental State Examination (MMSE), and categorized as impairment or maintenance, using previously specified cutoff points adjusted for education level [32].

To estimate physical activity level, the short form of the International Physical Activity Questionnaire (IPAQ) was applied [33]. Normal level of physical activity was defined for men and women as ≥ 383 and ≥ 270 kcal/week, respectively, and low level as < 383 and < 270 kcal/week, for men and women respectively [34].

Adherence to the Mediterranean diet (MeDi) was assessed by the Portuguese version of the Prevention with Mediterranean Diet tool (PREDIMED) [35]. Additionally, consumption of fish or shellfish ≥ 3 servings per week was assessed through a specific question included in PREDIMED: “How many servings of fish or shellfish do you consume per week? (1 serving: 100-150 g of fish or 4-5 units or 200 g of shellfish)” [36].

Health status was assessed by self-perceived health, which was categorized as follows: very good, good, moderate, bad and very bad [37].

Supplement intake concerning vitamin D and/or multivitamins containing vitamin D was self-reported by the participants. This includes participants that reported taking drugs containing vitamin D for osteoporosis treatment.

The Mini Nutritional Assessment® – short form tool (MNA®-SF) was applied to assess undernutrition status [38, 39].

Detailed anthropometric measurements were performed by the registered nutritionists. Body weight was measured with a calibrated scale (Seca 803, Germany), with a 0.1 kg resolution, and standing height with a stadiometer (Seca 213, Germany), with a 0.1 cm resolution, following standard procedures [40]. When necessary, weight was estimated from mid-upper arm and calf circumferences and height was estimated from nondominant hand length, as described elsewhere [20]. Circumferences were obtained with a metal tape (Lufkin, U.S.A., with 0.1 cm resolution) and hand length with a paquimeter (Fervi Equipment, Italy, with 0.1 cm resolution). Body mass index (BMI) was calculated using the standard formula [weight (kg)/height^2^ (m)]. According to BMI categories, participants were classified as underweight (< 18.5 kg/m^2^), normal range (18.5–24.9 kg/m^2^), overweight (25–29.9 kg/m^2^) and obese (≥ 30 kg/m^2^) [41].

Vitamin D status was assessed by dosing the serum 25(OH)D (nmol/L) concentrations. Blood collection occurred between December 2015 and June 2016, although the time period was not the same for all the districts. Therefore, blood collection occurred only for five or less months in some districts. All samples were analyzed in the same equipment (*Cobas Roche*) in one central laboratory (*General Lab*) in Portugal, by electrochemiluminescence assay using *Roche Diagnostic Vitamin D Total assay* (Roche Diagnostics GmbH, Mannheim, Germany) [42]. The detection limit of this test is 1.2 nmol/L [42].

### Exclusion criteria

Potential participants presenting any condition that disallowed the collection of venous blood or urine were excluded.

Subjects who had missing data regarding their location were excluded from the original sample.

### Statistical analysis

The mean of daily maximum UVI for the 30 days prior to the respective blood collection date (mUVI) was calculated for each participant. This time period was chosen for several reasons. In the literature, half-life of serum 25(OH)D has been reported to range from 2 to 3 weeks [43] to 2 months [44]. Also, the lag-time between a change in monthly UV dose and the corresponding change in 25(OH)D levels has been reported to range from 4 to 8 weeks [17, 45, 46].

The variable serum 25(OH)D concentrations (nmol/L) was treated as a continuous variable. Mean latitudes of the studied districts were calculated based on the participants’ postal code.

Data in descriptive statistics are presented as median and first and third quartile (Q1 and Q3) of 25(OH)D concentrations (due to a non-normal distribution) for each potential influential variable of serum 25(OH)D. For presentation of the results, variables were categorized as follows: age (65–69, 70–74, 75–79 and ≥ 80 years old), educational level (0, 1–4, 5–12 and ≥ 13 years of school completed), marital status (not married or married), household income (<500, 500–999, ≥ 1000 €/month and does not know or does not declare), skin phenotype (I-II, III-IV and V-VI), smoking habits (yes if at least 1 cigarette/month and no if < 1 cigarette/month) alcoholic beverages consumption (none, moderate if 1 drink/day for women and 1–2 drinks/day for men [47], and heavy if ≥ 2 drinks/day for women and ≥ 3 drinks/day for men), undernutrition status by MNA®-SF (without undernutrition and undernourished/at risk of undernutrition) and period of blood collection (December–March which comprises late autumn, winter and early spring, and April–June which comprises spring and early summer). This last categorization was also adopted by other authors [1, 48, 49].

In descriptive statistics UVI was treated as a categorical variable and it was categorized as low (1–2), moderate (3–5), high (6–7) and very high (8–10) [50].

According to 25(OH)D concentrations, participants were compared for several sociodemographic, lifestyle, health, nutritional and environmental characteristics. For the dichotomous variables, statistical significance of differences in serum 25(OH)D concentrations was assessed with the Mann-Whitney test. For variables with more than two categories, differences were tested using Kruskal-Wallis test and Mann-Whitney test with Bonferroni correction.

To illustrate the variation of serum 25(OH)D concentrations and mUVI, during the blood collection period, a chart was plotted. For each month, the median, Q1 and Q3 of 25(OH)D concentrations, as well as the mean and standard deviation (SD) of mUVI of the participants were calculated.

Mann-Whitney test with Bonferroni correction was performed to test statistical significance of differences in serum 25(OH)D concentrations between consecutive months.

The association between serum 25(OH)D concentrations and mUVI was explored by multiple linear regression analysis using the stepwise method. Due to a non-normal distribution of the dependent variable, 25(OH)D, a logarithm (log base 10) transformation was conducted [log_10_25(OH)D]. The following independent variables were included: mUVI (continuous), sex (dichotomous), age (continuous), education (categorical), professional activity (dichotomous), marital status (dichotomous), residence (dichotomous), household income (categorical), skin phenotype (categorical), cognitive performance (continuous), smoking habits (dichotomous), alcoholic beverages consumption (categorical), adherence to MeDi (continuous), fish or shellfish consumption ≥ 3 times/week (dichotomous), self-perceived health (categorical), supplement intake (dichotomous), undernutrition status by MNA®-SF (continuous) and BMI (continuous). Cognitive performance, measured by MMSE score, and undernutrition status, assessed by MNA®-SF score, were exponentially transformed before computing the model to achieve a normal distribution. Independent variables were chosen based on previous studies. Three multiple linear regression analyses were conducted using the same method. A regression analysis was conducted for the entire blood collection period, between December and June (Dec-Jun). Additionally, two regression analyses stratified by period of blood collection were conducted: between December and March (Dec-Mar) and between April and June (Apr-Jun).

For the results of stepwise linear regression analyses, standardized regression coefficients (sβ) and the respective 95% Confidence Interval (95% CI) are presented. Standardized regression coefficients were used to allow comparisons between explanatory variables [51].

## Results

### Descriptive statistics

Median 25(OH)D concentrations and other descriptive data, by potential influential factors of 25(OH)D, are shown in Table 1.

**Table 1:** Median 25(OH)D concentrations (nmol/L) of the sample, by potential influential factors

Variable	*n*	Median (Q1; Q3)	*P-value*
Sex			< 0.001
Female	872	32.95 (19.45; 52.88)	
Male	625	45.58 (26.10; 61.80)	
Age (years)			< 0.001
65–69	412	44.35 (29.95; 63.98)	
70–74	372	38.35 (25.60; 58.68)	
75–79	319	34.20 (20.20; 58.30)	
≥ 80	394	24.85 (14.50; 44.83)	
NUTS II			< 0.001
North	468	35.50 (20.35; 58.40)	
Center	391	31.50 (17.20; 50.80)	
Lisbon Metropolitan Area	383	42.20 (28.80; 62.30)	
Alentejo	136	30.60 (22.15; 51.48)	
Algarve	65	36.30 (20.75; 55.20)	
Madeira	30	54.45 (39.23; 74.85)	
Azores	24	42.75 (21.10; 59.88)	
Education (years)			< 0.001
0	212	23.80 (13.53; 35.20)	
1–4	1029	36.90 (21.95; 57.10)	
5–12	188	44.95 (29.18; 65.63)	
≥ 13	68	46.30 (31.18; 74.88)	
Professionally active			0.038
No	1462	35.75 (21.58; 57.15)	
Yes	30	46.55 (29.33; 65.60)	
Marital status			< 0.001
Single, divorced or widowed	796	29.85 (17.30; 48.20)	
Married or common-law marriage	700	43.40 (28.63; 64.10)	
Residence			< 0.001
Home	1425	36.80 (22.35; 58.00)	
Institution	72	17.90 (10.23; 38.28)	
Household income (€)			< 0.001
< 500	248	29.95 (17.48; 44.93)	
500–999	305	36.40 (24.15; 58.70)	
≥ 1000	174	48.55 (33.65; 69.88)	
Doesn’t declare/Doesn’t know	770	35.30 (19.90; 56.95)	
Skin phenotype			0.064
I + II	305	37.30 (24.85; 51.90)	
III + IV	1109	36.30 (21.30; 59.15)	
V + VI	80	30.00 (18.95; 46.33)	
Cognitive performance (MMSE)			< 0.001
Maintenance	1398	36.50 (22.10; 58.43)	
Impairement	99	28.10 (13.60; 45.90)	
Smoking habits			0.850
No	1429	35.90 (21.75; 57.40)	
Yes	68	36.15 (22.65; 51.83)	
Alcoholic beverages consumption			< 0.001
Does not drink	952	32.95 (20.33; 51.78)	
Moderate	388	43.85 (24.33; 64.63)	
Heavy	155	41.80 (27.80; 61.10)	
Physical activity (IPAC)			< 0.001
Normal	1234	38.30 (23.60; 59.00)	
Low	261	25.60 (14.85; 47.75)	
Adherence to Mediterranean diet (PREDIMED)			0.001
Low	849	34.20 (20.40; 54.70)	
High	648	39.05 (23.63; 60.30)	
Fish/shellfish consumption ≥ 3 servings/week			0.150
No	372	37.00 (23.70; 60.45)	
Yes	1125	35.50 (21.00; 56.30)	
Self-perceived health			< 0.001
Very good	69	44.30 (32.50; 64.20)	
Good	409	42.20 (24.40; 62.60)	
Moderate	730	35.30 (22.30; 57.10)	
Bad	232	28.30 (15.50; 44.23)	
Very bad	53	25.50 (17.25; 47.55)	
Supplement intake			<0.001
No	1369	34.90 (20.95; 54.85)	
Yes	128	54.55 (35.53; 82.78)	
Undernutrition status (MNA-SF)			<0.001
Without undernutrition	1256	37.50 (22.50; 58.88)	
Undernourished/at risk of undernutrition	241	29.00 (16.90; 47.65)	
Body mass index			<0.001
Underweight	3	38.20 (21.90; ^a^)	
Normal range	248	42.75 (23.25; 65.38)	
Overweight	660	38.55 (23.53; 60.60)	
Obese	582	31.20 (19.10; 50.10)	
mUVI categories			< 0.001
Low	570	30.05 (17.10; 48.50)	
Moderate	518	32.30 (19.88; 50.88)	
High	222	45.55 (29.88; 65.00)	
Very high	187	52.00 (39.00; 69.80)	
Period of blood collection			< 0.001
December – March	807	28.10 (16.70; 46.40)	
April – June	690	45.45 (30.40; 66.30)	

*n* number of subjects (does not always ad up to 1497 because of missing data). Q1: first quartile; Q3: third quartile

*P*-value for Mann-Whitney dichotomous variables or Kruskal-Wallis test for variables with > 2 categories

^a^Q3 was not possible to calculate

In this population of older adults, the median (Q1; Q3) of 25(OH)D concentration was 35.9 nmol/L (21.90; 57.35).

Analyzing median 25(OH)D concentrations for each variable (Table 1), these were significantly lower in women than in men, as well as in participants with ≥ 80 years old, no education, institutionalized, not professionally active, not married, with a household income of < 500 €/month, cognitively impaired, with no alcoholic beverages consumption, with low physical activity level, with low adherence to MeDi, that rated their health status as bad or very bad, without supplement intake, undernourished or at risk of undernutrition and whose blood was collected in December–March. Obese individuals had lower 25(OH) concentrations than subjects in the normal range or overweight (Mann-Whitney test *P* < 0.001).

Concentrations of serum 25(OH)D increased significantly across ascending mUVI categories (Mann-Whitney test after Bonferroni correction *P* < 0.001), except for the low and moderate categories (Mann-Whitney test after Bonferroni correction *P* > 0.008).

### Variation of mUVI and 25(OH)D by month of blood collection

**Fig. 1:**
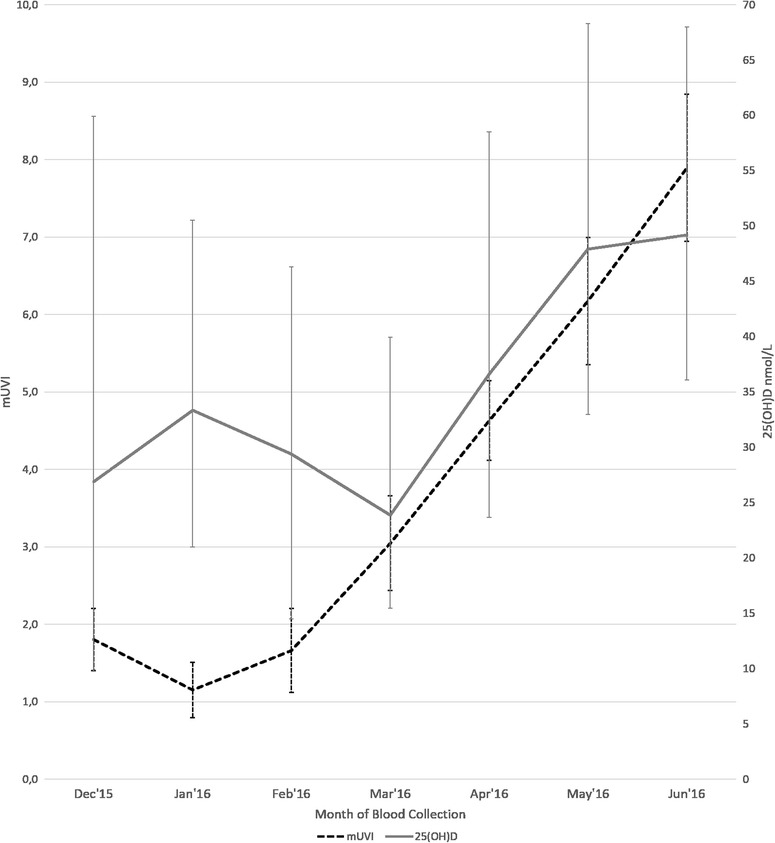
Variation of mUVI and 25(OH)D concentrations by month of blood collection. mUVI (mean values and SD in error bars) 25(OH)D concentrations (nmol/L) (median values, Q1 and Q3 in error bars)

The variation of mUVI and 25(OH)D concentrations during the blood collection period is shown in Fig. 1, where the mean of mUVI for all districts analysed in each month and the correspondent median 25(OH)D concentrations are presented. The mean of mUVI had its minimum in January (1.2) and increased until June, when it reached its peak (7.9). The mUVI was > 3 between April and June. Although 25(OH)D concentrations increased from December to January and median concentration reached its nadir in March (23.85 nmol/L), differences were not statistically significant between consecutive months within this period. After March 25(OH)D concentrations increased significantly between consecutive months until May (all *P* < 0.001), when the median concentration was not significantly different from June (49.2 nmol/L).

### Multiple linear regression analysis for the entire blood collection period

The model for Dec-June explained 28% of the variance in 25(OH)D concentrations (adjusted R^2^ = 0.28). Of all the continuous variables, mUVI had the highest effect on 25(OH)D concentrations (Sβ = 0.244).

In the model for Dec-Mar, mUVI was negatively associated with 25(OH)D concentrations (Sβ = −0.149), contrarily to the models for Apr-Jun (Sβ = 0.295) and Dec-June (Sβ = 0.244). Comparing models for Dec-Mar and Apr-Jun, some of the independent variables associated with 25(OH)D in the models were different. The following variables were associated in all three models: mUVI, age, supplement intake, undernutrition status and BMI. Both stratified models explained approximately 23% of the variance in 25(OH)D concentrations (Dec-Mar: adjusted R^2^ = 0.229; Apr-Jun: adjusted: R^2^ = 0.232).

In the multivariable model for Dec-Jun, an increase of 1 unit in mUVI was associated with an increase in 25(OH)D of 1.1 nmol/L (regression coefficient = 0.030). In multivariable the model for Apr-Jun an increase of 1 unit in mUVI was associated with an increase in 25(OH)D of 1.1 nmol/L (regression coefficient = 0.049).

## Discussion

In our cross-sectional study of Portuguese older adults, the median 25(OH)D concentration was 35.90 nmol/L.

In the Survey in Europe on Nutrition and the Elderly: A Concerted Action (EURONUT-SENECA study) the mean 25(OH)D levels ranged from 20 to 30 nmol/L in Southern European centers [52]. Comparisons should be taken cautiously due to discrepancies in latitude of the sample, ethnicity and season of blood collection [53].

In agreement with previous reports, the observed differences in 25(OH)D levels in our sample (Table 1), were as expected for the following variables: sex [53], age [53], physical activity [54, 55], BMI [48, 49], residence [56, 57] and supplement intake [23, 48].

The serum 25(OH)D concentrations increased significantly across ascending mUVI categories above moderate, which is in line with previous works [49, 54]. As expected, 25(OH)D concentrations were lower in Dec-Mar (late autumn-early spring) than in Apr-Jun (spring-early summer) which reflects the seasonal variation reported for 25(OH)D levels [48, 58] (Table 1).

Figure 1 showed a fluctuation of 25(OH)D concentrations and mUVI along the blood collection period. The variation of 25(OH)D from December to June was similar to previous studies, which have also found a minimum in March (or late winter/early spring) [59, 60] and an increase in spring and summer [1, 48, 61]. Similarly to another Portuguese work, frequency of sufficiency of vitamin D (≥ 75 nmol/L) was below 50% in every month [62]. It was noticeable that although mUVI started to increase in February, the rise of 25(OH)D concentrations only began in April, when mUVI was >3. This is in line with statements that UVI <3 does not trigger adequate synthesis of vitamin D [63, 64]. At latitudes >37°N, from November through February, the amount of UV radiation is usually not enough to initiate cutaneous synthesis [58]. O’Neill et al. (2016) also found that the UV threshold for adequate synthesis was only reached in mid-March, in European countries [59]. There might also have been a lag-time between a change on monthly UVI and the corresponding change in 25(OH)D levels, which has been reported to range from 4 to 8 weeks [17, 45, 46]. This lag-time may be linked to synthesis and half-life of vitamin D [65]. The decrease in 25(OH)D between January and March might have been influenced by the fact that UVI was not intense enough to trigger vitamin D synthesis [63, 64], and/or exposure was not likely to occur due to low temperatures, limited hours of sunshine and/or individual factors such as clothing [66–68].

The model for Dec-Jun (Table 2) explained 28% of the variance in 25(OH)D concentrations. [48, 54]. Associations between the independent variables and 25(OH)D were expected, according to previous research in middle aged and older populations, for: age [49, 54], household income [69], education [49], residence [70, 71], alcoholic beverages consumption [53, 72] physical activity [55, 73] self-perceived health [54], supplement intake [55, 74] and BMI [53, 54].

**Table 2:** Factors associated with 25(OH)D (nmol/L) by multiple linear regression for the period between December–June (*n* = 1486)

Independent variables	Sβ (95%CI)	*P value*
mUVI	0.244 (0.198; 0.291)	< 0.001
Age (years)	−0.135 (−0.184; −0.086)	< 0.001
Residence (home - 0; institution - 1)^a^	−0.064 (−0.110; −0.019)	0.005
Education (years)^b^		
0	−0.060 (−0.106; −0.014)	0.010
5–12	0.052 (0.007; 0.096)	0.022
Marital status (not married - 0; married - 1)^a^	0.089 (0.042; 0.137)	< 0.001
Household income (€ / month)^c^		
500–999	0.056 (0.011; 0.101)	0.015
≥ 1000	0.099 (0.052; 0.145)	< 0.001
Physical activity (normal - 0; low - 1)^a^	−0.078 (−0.124; −0.033)	0.001
Alcoholic beverages consumption - moderate ^d^	0.054 (0.011; 0.098)	0.015
Self-perceived health - bad ^e^	−0.051 (−0.096; −0.007)	0.024
Supplement intake (no - 0; yes - 1)^a^	0.202 (0.158; 0.245)	< 0.001
Undernutrition status (MNA®-SF score)	0.070 (0.025; 0.115)	0.002
BMI (kg/m^2^)	−0.123 (−0.168; −0.079)	< 0.001

*CI* confidence interval, *MNA®-SF* Mini Nutritional Assessment® – Short Form, *Sβ* Standardized regression coefficient

The model included only 1486 subjects because of missing data

^a^For the dichotomous variables, reference categories were coded as “0”

^b^Reference: “1–4 years”

^c^Reference: “Does not know or does not declare”

^d^Reference: “None”

^e^Reference: “Moderate”

Most studies that measured sun exposure or UV radiation availability found these were predictors of 25(OH)D concentrations [23, 49, 54]. The present study did not measure sun exposure, but used UVI as an indicator of potential for synthesis of vitamin D. The fact that mUVI was a predictor of 25(OH)D levels and that there was a positive association between the two is in line with other works that investigated the association between UVI and 25(OH)D levels [17–19]. However, Greer et al. (2013) found no correlation between the two variables, presumably to low sun exposure of the sample [28]. The four previous studies which have used mUVI and 25(OH)D concentrations did not perform an analysis separated in two seasons as was ran in this study [17–19, 28].

Undernutrition status, as measured by MNA®-SF score, was positively associated with 25(OH)D. A lower MNA®-SF score can be related to a decline in food intake and impaired mobility [39], which could discourage sun exposure and has been associated with vitamin D deficiency [56, 75].

Skin phenotype was not associated with 25(OH)D in the model, which occurred in some [76] but not all of the previous studies [49, 70]. This could have been influenced by the relative narrow range of skin types in our sample.

The majority of the previous studies concluded that dietary vitamin D intake [48, 49, 72] and fatty fish consumption [77] were predictors of 25(OH)D concentrations, which were not possible to estimate in our study. However, fish and shellfish consumption ≥ 3 servings/week and adherence to MeDi were not associated in the final model. The lack of discrimination between lean and fatty fish, inadequacy of their servings and the absence of questions linked to food with a high vitamin D content might have contributed to the absence of association found for these variables.

Stratification by period of blood collection resulted in models with different predictors of 25(OH)D and opposite associations between UVI and 25(OH)D (Tables 3 and 4).

**Table 3:** Factors associated with 25(OH)D by multiple linear regression for the period between December–March (*n* = 802)

December – March		
Independent variables	Sβ (95%CI)	*P-value*
mUVI	−0.149 (−0.211; −0.087)	< 0.001
Age (years)	−0.210 (−0.276; −0.146)	< 0.001
Residence	−0.104 (−0.169; −0.041)	0.001
Household income (€/month) - ≥1000	0.147 (0.082; 0.211)	< 0.001
Household income (€/month) - 500-999	0.069 (0.006; 0.132)	0.032
Alcoholic beverages consumption - Moderate	0.070 (0.009; 0.132)	0.025
Self-perceived health - Bad	−0.082 (−0.144; −0.020)	0.010
Supplement intake	0.196 (0.133; 0.256)	< 0.001
Undernutrition status (MNA-SF score)	0.097 (0.034; 0.159)	0.003
BMI (kg/m^2^)	−0.164 (−0.226; −0.102)	<0.001

*CI* confidence interval, *MNA®-SF* Mini Nutritional Assessment® – Short Form, *Sβ* Standardized regression coefficient

**Table 4:** Factors associated with 25(OH)D by multiple linear regression for the period between April–June (*n* = 683)

April–June (n = 683)		
Independent variables	Sβ (95%CI)	*P-value*
mUVI	0.295 (0.229; 0.362)	< 0.001
Age (years)	−0.092 (−0.163; −0.022)	0.010
Education (years) - zero	−0.078 (−0.148; −0.010)	0.025
Marital Status	0.140 (0.072; 0.209)	< 0.001
Skin phenotype - V + VI	−0.079 (−0.145; −0.013)	0.019
Cognitive performance (MMSE score)	0.086 (0.016; 0.156)	0.016
Physical Activity - Low	−0.102 (−0.172; −0.034)	0.004
Supplement intake	0.246 (0.181; 0.315)	< 0.001
Undernutrition status (MNA-SF score)	0.076 (0.008; 0.145)	0.030
BMI (kg/m^2^)	−0.117 (−0.185; −0.050)	0.001

*CI* confidence interval, *MNA®-SF* Mini Nutritional Assessment® – Short Form, *Sβ* Standardized regression coefficient

In the model for Dec-Mar, mUVI was inversely associated with 25(OH)D. This association was expected because, as shown in Fig. 1, between Dec-Mar 25(OH)D declined despite mUVI was rising. Factors that have been previously discussed for Fig. 1 and were observed in other studies could have contributed to this negative association [66, 67, 78]. The model for Dec-Jun showed a smaller effect of mUVI upon 25(OH)D concentrations than the model for Apr-Jun. This difference could be explained by the lack of effect of mUVI during the Dec-Mar period, in which the average mUVI values are below the threshold for vitamin D synthesis. Nevertheless, we considered this model for comparison with other published studies.

The present study shows that given the Portuguese latitudes, UV may not be high enough to trigger vitamin D synthesis between late autumn and early spring [8, 11–13]. During this period individuals may have to rely on their vitamin D reserves, which may not last all “vitamin D winter”, on diet and on supplements [79]. Since vitamin D ingestion may not be adequate, supplementation may be advisable in older adults [63, 68].

In the model for Apr-Jun**,** mUVI was positively associated with 25(OH)D, which is in line with previous studies [17–19]. In these months sun exposure is more likely to occur [23] and it was expected that the higher UVI (mUVI > 3) would promote synthesis and contribute to the higher 25(OH)D concentrations, comparatively to Dec-Mar [63, 80]. The observation of low 25(OH)D concentrations despite UVI > 3 is in line with reports of high levels of deficiency even in regions with high UVI, particularly in risk groups as the elderly [67, 81]. Additionally, older people synthesize vitamin D less efficiently [63] and tend to avoid sun exposure even when temperatures are high [21]. The widespread public health advice on skin protection can also contribute to limited sun exposure [3].

The fact that different predictors have been selected in both stratified models and UVI associations were opposite may also be related with the characteristics of the different groups of participants.

Nevertheless, UV exposure can increase 25(OH)D in older adults, depending on the season [82, 83]. Moreover, sun exposure during summer is a major determinant for vitamin D stores [84]. Therefore, even though deficiency was frequent in this and other works, optimizing vitamin D stores is still important to maintain or at least to minimize the decline of vitamin D status during winter for as long as possible [15, 53].

As far as we are concerned, this is the first study to explore the relationship between UVI and 25(OH)D in older people in Portugal. Also, only four studies could be found to explore this relationship in other countries [17–19, 28]. The main strengths of this study include the large sample size, the nationwide coverage and the fact that the assay, equipment and laboratory of 25(OH)D quantification were the same, which decreased the variability existent in several works [1]. Moreover, the model for Dec-Jun explained 28% of the variance in 25(OH)D concentrations, which was in line with the results from previous studies that included sun exposure, diet and genetic factors as potential predictors of 25(OH)D concentrations. The use of UVI as an indicator of potential for vitamin D synthesis, instead of latitude and season, constitutes another strength of this study, since UVI accounts for latitude, season and intensity of UV that reaches the Earth adjusted for nebulosity and ozone absorption [79].

### Limitations

The UVI is based on the UV spectrum for erythema, which some authors argue that diverges from the vitamin D action spectrum [85].

It was not possible to estimate sun exposure from the collected data and UVI may indicate only the potential to synthesize vitamin D. Various factors that were not possible to assess may have interfered in this potential, including sun exposure behaviors (such as sunscreen use, clothing, duration and location of exposure) and ambient factors such as pollution [86]. Genetic factors, interindividual variability, diet, certain medications, chronic kidney conditions, including renal failure, and other diseases may also have affected 25(OH)D synthesis and/or concentrations of our sample but is effect was not accounted for.

The measured 25(OH)D concentrations may not reflect the long-term levels of the population [81]. The fact that this is a cross-sectional observational study does not allow the establishment of causal relationships. The sample of our study was not randomly selected and a participation bias might have existed, which impair the generalization of our results to the population.

## Conclusions

This study shows that the association between UVI and vitamin D may be different depending on the time of the year and thus, future research and recommendations about sensible sun exposure and vitamin D status should take this into account. Diet and supplements may be more important D during months when UVI and sun exposure are low/insufficient and should be reinforced accordingly.

## Additional files

Additional file 1: Portuguese questionnaire applied to participants. File containing the full questionnaire that was applied to all participants. (PDF 346 kb)

Additional file 2: An English translation of the Portuguese questionnaire applied to participants. File containing the translated questionnaire that was applied to all participants. (PDF 1005 kb)

## Abbreviations

25(OH)D: 25-hydroxitamin D
BMI: Body Mass Index
EURONUT-SENECA: Survey in Europe on Nutrition and the Elderly: A Concerted Action
IOM: Institute of Medicine
IPAC: International Physical Activity Questionnaire
MeDi: Mediterranean diet
MET: Metabolic equivalent task
MMSE: Mini Mental State Examination
MNA®-SF: Mini Nutritional Assessment Short Form
mUVI: Mean Ultraviolet Index
NUTS II: Nomenclature of Territorial Units for Statistics II
PREDIMED: Prevention with Mediterranean Diet tool
UVB: Ultraviolet B
UVI: Ultraviolet Index
