# Supplementary material for: Association between serum 25-hidroxyvitamin D concentrations and ultraviolet index in Portuguese older adults: a cross-sectional study
Source: BMC Geriatr. 2017 Oct 31;17:256. doi: 10.1186/s12877-017-0644-8 (PMC5664428; doi:10.1186/s12877-017-0644-8)
Supplement: Supplementary file 1 — Portuguese questionnaire applied to participants. File containing the full questionnaire that was applied to all participants. (PDF 346 kb) [file 12877_2017_644_MOESM1_ESM.pdf]

Código do entrevistador:	|__|__|
Área regional:	|__|
Tipologia de local de residência:	Domicílio |__|(0)<br>Instituição:<br>Lar |__|(1)<br>Outro |__|(2) Especificar: ______________________
Nº identificação:	|__|__|__|__|
Data:	|___|(Dia) - |___|(Mês) - |___|(Ano)

1. CARATERIZAÇÃO SÓCIO-DEMOGRÁFICA

1.1. Sexo F|__| (0) M |__| (1)

1.2. A sua data de nascimento é: |__|__| (Dia) | __|__| (Mês) | __|__| (Ano) Não sabe: |__| (9)

A sua idade é: |__|__|__| (Anos)

1.3. Qual é o seu estado marital? *Informação que consta no registo civil ou outra*

Solteiro(a) |__| (0)

Casado(a) |__| (1)

Divorciado (a) |__| (2)

União de facto |__| (3)

Viúvo(a) |__| (4)

Prefere não responder |__| (8)

Não sabe |__| (9)

1.4 Qual é o seu nível de escolaridade? Isto é, quantos anos completos de escolaridade, com aproveitamento, frequentou?

Caso não consiga identificar o seu nível de escolaridade, escolha a opção "Sem correspondência (anos/níveis antigos)". Se o nível de escolaridade mais elevado que completou foi obtido no estrangeiro, assinale o nível correspondente no sistema de ensino português.

Nenhum|__| (0)

1.ª ou 2.ª ou 3.ª classes completas |__| (1)

4ª classe completa|__| (2)

Liceu –1º, 2º, 3º ou 4º ano completos|__| (3)

Liceu – 5º ano completo|__| (4)

Liceu - 6º ou 7º anos completos |__| (5)

Pós-secundário, ou seja, cursos de especialização tecnológica não superior |__| (6)

Superior – bacharelato |__| (7)

Superior – licenciatura |__| (8)

Superior – mestrado |__| (9)

Superior – doutoramento |__| (10)

Sem correspondência |__| (11)

Prefere não responder |__| (98)

Não sabe| __| (99)

2. MINI MENTAL STATE EXAMINATION (MMSE)

Atenção défice cognitivo se: *analfabetos ≤ 15, 1 a 11 anos completos de escolaridade ≤ 22 e > 11 anos de escolaridade ≤ 27*

- Se o participante obtiver pontuação indicativa de defeito cognitivo ou não conseguir assinar o consentimento informado, obter a informação através do seu tutor legal ou a assinatura de duas testemunhas e a sua identificação (nome e cartão de cidadão).

2.1. Orientação *(1 ponto por cada resposta correta)*

2.1.1. Em que ano estamos? |__| 2.1.2. Em que mês estamos? |__| 2.1.3. Em que dia do mês estamos? |__|

2.1.4. Em que dia da semana estamos? |__| 2.1.5. Em que estação do ano estamos? |__|

2.1.6. Em que país estamos? |__| 2.1.7. Em que distrito vive? |__| 2.1.8. Em que terra vive? |__|

2.1.9. Em que casa estamos? |__| 2.1.10. Em que andar estamos? |__| **2.1.11. Nota:** |__|

2.2. Retenção *(1 ponto por cada palavra corretamente repetida)*

"Vou dizer três palavras; queria que as repetisse, mas só depois de eu as dizer todas; procure ficar a sabê-las de cor".

- Pera - Gato - Bola **Nota:** |__|

2.3. Atenção e Cálculo *(1 ponto por cada resposta correta. Se der uma errada mas depois continuar a subtrair bem, consideram-se as seguintes como corretas. Parar ao fim de 5 respostas)*

"Agora peço-lhe que me diga quantos são 30 menos 3 e depois ao número encontrado volta a tirar 3 e repete assim até eu lhe dizer para parar".

27 |__| **24** |__| **21** |__| **18** |__| **15** |__| Nota: |__|

2.4. Evocação *(1 ponto por cada resposta correta.)*

"Veja se consegue dizer as três palavras que pedi há pouco para decorar".

- Pera - Gato - Bola **Nota:** |__|

2.5. Linguagem *(1 ponto por cada resposta correta)*

"Como se chama isto? Mostrar os objetos:

a. Relógio |__| b. Lápis |__| **Nota:** |__|

c. "Repita a frase que eu vou dizer: O RATO ROEU A ROLHA" **Nota:** |__|

d. "Quando eu lhe der esta folha de papel, pegue nela com a mão direita, dobre-a ao meio e ponha sobre a mesa"; dar a folha segurando com as duas mãos.

- Pega com a mão direita |__| - Dobra ao meio |__| - Coloca onde deve|__| **Nota:** |__|

e. "Leia o que está neste cartão e faça o que lá diz". Mostrar um cartão com a frase bem legível, "FECHE OS OLHOS"; sendo analfabeto lê-se a frase.

- Fechou os olhos **Nota:** |__|

f. "Escreva uma frase inteira aqui". Deve ter sujeito e verbo e fazer sentido; os erros gramaticais não prejudicam a pontuação.

Frase: ____ **Nota:** |__|

2.6. Habilidade Construtiva *(1 ponto pela cópia correta.)*

Deve copiar um desenho. Dois pentágonos parcialmente sobrepostos; cada um deve ficar com 5 lados, dois dos quais intersectados. Não valorizar tremor ou rotação.

Cópia:

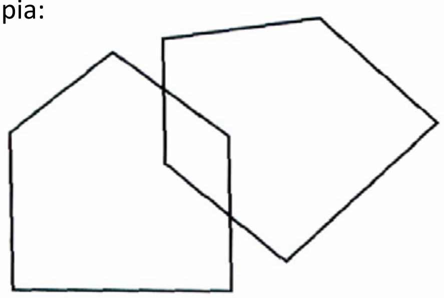

Nota: |__|

2.7 Nota total (máximo 30 pontos): |__|__|

<u>**Considera-se com comprometimento cognitivo**</u>: *s/escolaridade ≤ 15, 1 a 11 anos de escolaridade ≤ 22 e > 11 anos de escolaridade ≤ 27*

- Se o participante obtiver pontuação indicativa de defeito cognitivo ou não conseguir assinar o consentimento informado, obter a informação através do seu tutor legal ou a assinatura de duas testemunhas e a sua identificação (nome e cartão de cidadão).

3. QUEM FORNECE INFORMAÇÃO

O próprio |__| (0) Um familiar |__| (1) Outra pessoa |__| (2)

4. ATUALMENTE, COMO SE ENCONTRA EM TERMOS PROFISSIONAIS?

Ativo|__| (0) Se ativo passar para a questão **4.1.**

Não ativo |__| (1) Se não ativo passar para a questão **4.2.**

4.1. Tem um emprego ou trabalho.

4.1.1. Se trabalha, qual a sua profissão principal?
- Escolha também esta opção se trabalha sem ser pago para um familiar com quem vive.
- Indique a sua profissão da forma mais completa possível ou descreva as principais tarefas que executa.
- Se tiver mais do que uma profissão, responda em relação aquela que atualmente lhe ocupa mais tempo (maior número de horas).
Descritivo da profissão:

____

____

4.1.2. Trabalha a tempo inteiro?

Sim |__| (0) Não |__| (1)

Prefere não responder |__| (8)

4.2. *Escolha esta opção apenas se teve um emprego/trabalho e agora está reformado. Se nunca trabalhou, mas recebe uma reforma ou pensão escolha a opção "**Tem outra situação de inatividade".***

Está reformado?|__|(0) ou com reforma antecipada? |__| (1) está desempregado |__| (2)

Outra situação de inatividade |__|(3)

4.2.1. Qual foi a sua profissão principal?
- Indique a sua profissão da forma mais completa possível ou descreva as principais tarefas que executou. Se tiver tido mais do que uma profissão, responda em relação aquela que lhe ocupou mais tempo (maior número de horas).
Descritivo da profissão:

____

____

4.3. Outras Ocupações

Ocupa-se de tarefas domésticas |__| (0)

Presta serviço cívico ou comunitário (obrigatório ou voluntário) |__| (1)

Prefere não responder |__| (8)

Não sabe |__| (9)

5. ESTILOS DE VIDA

5.1 ATIVIDADE/EXERCÍCIO FÍSICO

- Estamos interessados em conhecer os diferentes tipos de atividade física, que as pessoas fazem no seu quotidiano. As questões que lhe vou colocar, referem-se à semana imediatamente anterior, considerando o tempo em que esteve fisicamente ativo/a. Por favor, responda a todas as questões, mesmo que não se considere uma pessoa fisicamente ativa. Vamos colocar-lhe questões sobre as atividades desenvolvidas na sua atividade profissional e nas suas deslocações, sobre as atividades referentes aos trabalhos domésticos e às atividades que efetuou no seu tempo livre para recreação ou prática de exercício físico / desporto.

Ao responder às seguintes questões considere o seguinte:

*- **Atividades Físicas Vigorosas:** referem-se as atividades que requerem um esforço físico intenso que fazem ficar com a respiração ofegante.*
*- **Atividades Físicas Moderadas:** referem-se as atividades que requerem esforço físico moderado e tornam a respiração um pouco mais forte que o normal.*
- Ao responder às questões considere apenas as atividades físicas que realize durante pelo menos 10 minutos seguidos.

5.1.1. Diga-me por favor, nos últimos 7 dias, em quantos dias andou/caminhou pelo menos 10 minutos seguidos?

N.º |__|dias

5.1.2. Quanto, tempo no total, despendeu num desses dias, a andar/caminhar?

|__|__|horas |__|__|minutos

5.1.3. Diga-me por favor, num dia normal, quanto tempo passa sentado? Isto pode incluir o tempo que passa a uma secretária, a visitar amigos, a ler, a estudar ou a ver televisão.

|__|__|Horas |__|__|minutos

5.1.4. Diga-me por favor, nos últimos 7 dias, em quantos dias fez atividades físicas vigorosas, como por exemplo, levantar objetos pesados, agricultura, cavar, ginástica aeróbica, nadar, jogar futebol, andar de bicicleta a um ritmo rápido?

N.º |__|dias

5.1.5. Nos dias em que pratica atividades físicas vigorosas, quanto tempo em média dedica normalmente a essas atividades por dia?

|__|__|Horas |__|__|minutos

5.1.6. Diga-me por favor, nos últimos 7 dias, em quantos dias fez atividades físicas moderadas como por exemplo, carregar objetos leves, caçar, trabalhos de carpintaria, jardinagem, andar de bicicleta a um ritmo normal ou ténis de pares? Por favor não inclua o "andar".

N.º |__|dias

5.1.7. Nos dias em que pratica atividades físicas moderadas, quanto tempo em média dedica normalmente a essas atividades por dia?

|__|__|Horas |__|__|minutos

5.2 CONSUMO DE TABACO

Considere qualquer tipo de tabaco, com exceção do cigarro eletrónico

5.2.1. Atualmente fuma?

Não|__| (0) Sim |__| (1) Prefere não responder |__| (8)

5.2.2. Com que frequência fuma?

Diariamente |__|(0) Semanalmente |__|(1) Mensalmente ou com menor frequência |__|(2)

5.2.3. *Para quem reporta fumar diariamente:*

Em média quantos cigarros fuma por dia (n.º cigarros)? |__|__|__|

5.3. CONSOME BEBIDAS ALCOÓLICAS?

5.3.1. Não |__| (0) Sim |__| (1): Prefere não responder |__| (8)

5.3.2. Com que frequência bebe?

Todos os dias|__| (0) Duas a seis vezes por semana |__| (1) Uma vez por semana |__|(2)

Menos de uma vez por semana |__| (3)

5.3.3. Em média, quantas bebidas ingere num dia em que consuma bebidas alcoólicas? |__|__|

Prefere não responder |__| (88)

5.4. ADESÃO AO PADRÃO ALIMENTAR DO TIPO MEDITERRÂNICO *(PREDIMED)*

		Critérios para atribuir 1 ponto	Pontuação
1	Utiliza azeite como principal gordura culinária?	Sim	
2	Que quantidade de azeite consome num dia (incluindo uso para fritar, temperar saladas, refeições fora de casa, etc.)?	≥ 4 colheres sopa	
3	Quantas porções de produtos hortícolas consome por dia? *(1 porção: 200 g; considere acompanhamentos como metade de uma porção)*	≥ 2 porções por dia (ou ≥1 porção crua ou em salada)	
4	Quantas peças de fruta (incluindo sumos de fruta natural) consome por dia?	≥ 3 por dia	
5	Quantas porções de carne vermelha, hambúrguer ou produtos cárneos (presunto, salsicha, etc.) consome por dia? *(1 porção: 100-150 g)*	< 1 porção por dia	
6	Quantas porções de manteiga, margarina, ou natas consome por dia? *(1 porção: 12 g)*	< 1 porção por dia	
7	Quantas bebidas açucaradas ou gaseificadas bebe por dia?	<1 por dia	
8	Quantos copos de vinho bebe por semana?	≥ 7 copos por semana	
9	Quantas porções de leguminosas consome por semana? *(1 porção: 150 g)*	≥ 3 por semana	
10	Quantas porções de peixe ou marisco consome por semana? *(1 porção: 100-150 g de peixe ou 4-5 unidades ou 200 g de marisco)*	≥3 por semana	
11	Quantas vezes por semana consome produtos de pastelaria ou doces comerciais (não caseiros), como bolos, bolachas, biscoitos?	<3 vezes por semana	
12	Quantas porções de oleaginosas (nozes, amêndoas, incluindo amendoins) consome por semana? *(1 porção 30 g)*	≥ 3 por semana	
13	Consome preferencialmente frango, peru ou coelho em vez de vaca, porco, hambúrguer ou salsicha?	Sim	
14	Quantas vezes por semana consome hortícolas, massa, arroz ou outros pratos confecionados com um refogado (molho à base de tomate, cebola, alho-francês ou alho e azeite)?	≥ 2 vezes por semana	
**Boa adesão à dieta mediterrânica**		**Pontuação Final ≥ 10**	

Traduzido e adaptado por Afonso et al, 2014 de Martínez-Gonzales et al, 2012

6. ESTADO DE SAÚDE

6.1 PERCEÇÃO SOBRE O ESTADO DE SAÚDE

- A pergunta seguinte refere-se à perceção do seu estado de saúde.

- Responda à pergunta escolhendo a resposta mais adequada. Se não tiver a certeza como responder, por favor dê a resposta que lhe parecer mais apropriada

De uma maneira geral, como considera o seu estado de saúde?

Muito bom |__| (0)
Bom |__| (1)
Razoável |__| (2)
Mau |__| (3)
Muito mau |__| (4)
Prefere não responder |__| (8)
Não sabe |__| (9)

7. DOENÇAS CRÓNICAS

7.1. Tem alguma doença crónica ou problema de saúde prolongado?

- Responda "Sim" se o problema de saúde dura ou possa vir a durar mais de 6 meses. Considere os problemas de saúde controlados com medicação, problemas sazonais (p. ex. alergias) ou problemas de saúde causados por lesões, patologias congénitas ou malformações à nascença.

Não |__| (0) Sim |__| (1) Prefere não responder |__| (8)

7.2. Indique se, durante os últimos 12 meses, sofreu das seguintes doenças:

	Não (0)	Sim (1)	Prefere não responder (8)	Não sabe (9)
**7.2.1.** Asma (incluindo asma alérgica)				
**7.2.2.** Bronquite crónica, doença pulmonar obstrutiva crónica ou enfisema				
**7.2.3**. Enfarte do miocárdio (ou ataque cardíaco) ou de consequências crónicas de um enfarte do miocárdio				
**7.2.4.** Doença coronária ou angina de peito (*Considere todas as doenças das artérias do coração. Não considere as consequências crónicas do enfarte do miocárdio)*				
**7.2.5.**Tensão arterial alta, *i.e.*, hipertensão arterial				
**7.2.6.** AVC (acidente vascular cerebral) ou de consequências crónicas de um AVC				
**7.2.7.** Artrose (ou doença degenerativa das articulações)				
**7.2.8.** Dores lombares ou outros problemas crónicos nas costas				
**7.2.9.** Dores cervicais ou outros problemas crónicos no pescoço				
**7.2.10.** Diabetes				
**7.2.11.** Cirrose hepática<br>*Doença crónica do fígado. Inclui todas as cirroses (mesmo não alcoólicas)*				
**7.2.12.** Alergias (rinite, febre dos fenos, conjuntivite alérgica, dermatite, alergias alimentares ou outras alergias)<br>*Não considere a asma alérgica*				
**7.2.13.** Problemas renais crónicos, incluindo insuficiência renal<br>*As pedras nos rins só devem ser consideradas caso entenda que é um problema crónico ou prolongado*				
**7.2.14.** Incontinência urinária ou problemas de controlo da bexiga				
**7.2.15.** Depressão				
**7.2.16.** Outra? Se sim, registar o nome	____________________________________			

7.3. REGISTO DE MEDICAMENTOS e SUPLEMENTOS NUTRICIONAIS

Atualmente, que medicamentos e suplementos nutricionais toma diariamente? - *Registar o nome e tomas diárias.*

Nome do **medicamento**		Nº de tomas diárias	Nome do **suplemento nutricional**		Nº de tomas diárias
**1**			**1**		
**2**			**2**		
**3**			**3**		
**4**			**4**		
**5**			**5**		
**6**			**6**		
**7**			**7**		
**8**			**8**		
**9**			**9**		
**10**			**10**		

8. ESTADO NUTRICIONAL

8.1 ANTROPOMETRIA

8.1.1. Peso *- Registe o peso com roupas leves e sem sapatos* |__|__|__| , |__|(kg)

8.1.2. Estatura *- Registe a estatura sem sapatos* |__|__|__| , |__| (cm)

- Se não puder medir, meça o: Comprimento da mão: |__|__| , |__| (cm)

8.1.3. Perímetro do meio braço |__|__|__| , |__| (cm)

8.1.4. Perímetro da cintura |__|__|__| , |__| (cm)

8.1.5. Perímetro geminal |__|__|__| , |__| (cm)

8.1.6. Prega cutânea tricipital |__|__| , |__| (mm)

8.2. INDICADORES FUNCIONAIS

8.2.1. Força preensora da mão

1ª medição |__|__|__| , |__| (kgf)

2ª medição |__|__|__| , |__| (kgf)

3ª medição |__|__|__| , |__| (kgf)

8.2.2. Velocidade da passada *(tempo necessário para caminhar 4,6m)* |__|__|__| , |__| (seg)

8.2.3. *Mini-nutritional assessment - Short Form*[a]
- Completar a avaliação, preenchendo as caixas com os números adequados. Somar para obter o score final de rastreio.

A. Nos últimos três meses houve diminuição da ingestão alimentar devido à perda de apetite, problemas digestivos ou dificuldade para mastigar ou deglutir? |__|
0 = diminuição grave da ingestão
1 = diminuição moderada da ingestão
2 = sem diminuição da ingestão

B. Perda de peso nos últimos 3 meses |__|
0 = superior a três quilos
1 = não sabe informar
2 = entre um e três quilos
3 = sem perda de peso

C. Mobilidade |__|
0 = restrito ao leito ou à cadeira de rodas
1 = deambula mas não é capaz de sair de casa
2 = normal

D. Passou por algum estresse psicológico ou doença aguda nos últimos três meses? |__|
0 = sim
2 = não

E. Problemas neuropsicológicos |__|
0 = demência ou depressão graves
1 = demência leve
2 = sem problemas psicológicos

F1. Índice de Massa Corporal (IMC = peso [kg] / estatura^2 [m]) |__|
0 = IMC < 19
1 = 19 ≤ IMC < 21
2 = 21 ≤ IMC < 23
3 = IMC ≥ 23

[a]Traduzido e adaptado de: Vellas B *et al*. *Overview of the MNA® - Its History and Challenges*. J Nutr Health Aging 2006;10:456-465. Rubenstein LZ *et al*. *Screening for Undernutrition in Geriatric Practice: Developing the Short-Form Mini Nutritional Assessment (MNA-SF)*. J. Geront 2001;56A: M366-377.)

- SE O CÁLCULO DO IMC NÃO FOR POSSÍVEL, SUBSTITUIR A QUESTÃO F1 PELA F2.
NÃO PREENCHA A QUESTÃO F2 SE A QUESTÃO F1 JÁ TIVER SIDO COMPLETADA.

F2. Perímetro Geminal (PG) em cm |__|
0 = PG menor que 31
3 = PG maior ou igual a 31

- 12-14 pontos: estado nutricional normal
- 8-11 pontos: sob risco de desnutrição
- 0-7 pontos: desnutrido

Score de Rastreio (máximo: 14 pontos) |__|__|

8.2.4. FRAGILIDADE - ESCALA DE FRIED

8.2.4.1. PERDA DE PESO

>4,5 kg no último ano, de forma não intencional — Não |__| (0) Sim |__| (1)
Não sabe |__| (9)

8.2.5. EXAUSTÃO

8.2.5.1. "Tudo o que faço é em esforço" Na semana passada, quantas vezes se sentiu assim? |__|__|

8.2.5.2. “Eu não consigo continuar"

Nunca ou quase nunca (< 1 dia) |__| (0)
Às vezes (1 – 2 dias) |__| (1)
Algumas vezes (3 – 4 dias) |__| (2)
Quase sempre ou sempre |__| (3)

9. COABITAÇÃO

- Diga-me, por favor com quem vive:

Grau de parentesco	Sexo	Idade	Grau de parentesco	Sexo	Idade

10. FENÓTIPO CUTÂNEO

Qual é o seu tipo de pele?

a. Pele clara, olhos azuis, sardentos (sempre se queima e nunca se bronzeia) |__| (0)
b. Pele clara, olhos azuis, verdes ou castanhos-claros, cabelos louros ou ruivos (sempre se queima e, às vezes, bronzeia) |__| (1)
c. Como a média das peles brancas normais (queima-se moderadamente, bronzeia-se gradual e uniformemente) |__| (2)
d. Pele clara ou morena clara, cabelos castanhos-escuros e olhos escuros (queima-se muito pouco, bronzeia-se bastante) |__| (3)
e. Pele morena (raramente se queima, bronzeia-se muito) |__| (4)
f. Pele negra (nunca se queima, profundamente pigmentada). |__| (5)

11. E para terminar, pedia que me indicasse o valor aproximado do rendimento mensal total do seu agregado familiar (considerando todas as fontes de rendimento), líquido, depois de deduzir todos os impostos.

|__|__|__||__|__|__|€ — Prefere não responder |__| (8)

INFORMAÇÃO AO PARTICIPANTE

O projeto ***"Nutrição para maiores de 65|estratégias nutricionais face ao envelhecimento"*** está a ser conduzido pela Faculdade de Ciências da Nutrição e Alimentação da Universidade do Porto.

Este estudo tem como objetivo conhecer o estado nutricional das pessoas idosas portuguesas, especificamente sobre a desnutrição, obesidade, sarcopenia, fragilidade, estado de vitamina D, de hidratação e a excreção urinária de sódio e de potássio. Terá como principal benefício por participar neste estudo, a possibilidade de conhecer os seus resultados relativamente a estes exames efetuados. Os seus resultados e os de os outros participantes neste estudo permitirão também desenvolver programas de educação alimentar adaptados a este grupo.

Iremos pedir-lhe que responda a questões cuja informação é importante para o estudo. Todos os procedimentos são simples e serão realizados por profissionais previamente treinados para o efeito.

Não estão previstos potenciais riscos e desconfortos relativos à sua participação, a não ser os resultantes da recolha da amostra de sangue e de urina, da avaliação do peso, de perímetros do braço e da perna, da altura, da prega de gordura, da força muscular da mão e da sua velocidade da passada. Será efetuado um questionário que terá a duração de cerca de 20 minutos.

Toda a informação que nos fornecer é confidencial e a privacidade da mesma será sempre garantida.

Informamos que a sua participação **é voluntária, tendo o direito de recusar ou de desistir, sem que isso possa ter como efeito qualquer prejuízo pessoal.**

Agradecemos, desde já, toda a atenção dispensada.

Contacto do Investigador Responsável:
Xxxxxxxxx (Daily Spin) Telefone: xxxxxxx
FCNAUP, Telefone: 225074320

DECLARAÇÃO DE CONSENTIMENTO

Considerando a última versão da "Declaração de Helsínquia" da Associação Médica Mundial

Nutrição para maiores de 65| estratégias nutricionais face ao envelhecimento demográfico

Eu, abaixo-assinado, ____,

declaro ter compreendido a explicação que me foi fornecida acerca do estudo que se tenciona realizar. Foi-me dada a oportunidade de fazer as perguntas que julguei necessárias e de todas obtive uma resposta satisfatória.

Tomei conhecimento de que, de acordo com as recomendações da Declaração de Helsínquia, a informação ou explicação que me foi prestada versou os objetivos, os métodos, os benefícios previstos, os riscos potenciais e o eventual desconforto. Além disso, foi-me afirmado que tenho o direito de recusar a todo o tempo a minha participação no estudo, sem que isso possa ter como efeito qualquer prejuízo pessoal.

Por isso, consinto que me sejam aplicados os procedimentos propostos pelo investigador.

Data: _____ / __________________ / 201___

Assinatura do voluntário:

____

Assinatura do investigador responsável:

____
