# Supplementary material for: Association between serum 25-hidroxyvitamin D concentrations and ultraviolet index in Portuguese older adults: a cross-sectional study
Source: BMC Geriatr. 2017 Oct 31;17:256. doi: 10.1186/s12877-017-0644-8 (PMC5664428; doi:10.1186/s12877-017-0644-8)
Supplement: Supplementary file 2 — An English translation of the Portuguese questionnaire applied to participants. File containing the translated questionnaire that was applied to all participants. (PDF 1005 kb) [file 12877_2017_644_MOESM2_ESM.pdf]

Interviewer Code:	|__|__|
Regional area:	|__|
Type of residence:	Home|__|(0)<br>Institution:<br>Retirement home|__|(1)<br>Other|__|(2) Specify ______________________
Identification number:	|__|__|__|__|
Date:	|___|(Day) - |___|(Month) - |___|(Year)

1. SOCIODEMOGRAPHIC CHARACTERIZATION

1.1. Sex F|__| (0) M |__| (1)

1.2. Your date of birth is: |__|__| (Day) | __|__| (Month) | __|__| (Year) Do not know: |__| (9)

Your age is: |__|__|__| (Years)

1.3. What is your marital status? *Information that appears in the civil registry or other*

Single |__| (0)

Married |__| (1)

Divorced |__| (2)

Common-law marriage |__| (3)

Widowed |__| (4)

Prefer not to answer |__| (8)

Do not know |__| (9)

1.4 What is your level of education? That is, how many full years of schooling did you attend?
If you cannot identify your level of education, choose the option "No matching (years/old levels)". If the highest level of education completed was obtained abroad, please note the corresponding level in the Portuguese education system.
None|__| (0)

Elementary school - 1st or 2nd or 3rd grades completed |__| (1)

Elementary school - 4th grade completed |__| (2)

High School - 1st or 2nd or 3rd or 4th grades completed |__| (3)

High School - 5th grade completed |__| (4)

High School - 6th or 7th grades completed |__| (5)

Post-secondary, that is, courses of technological specialization not superior |__| (6)

Post-secondary – technical specialization |__| (7)

Higher education - bachelor's degree |__| (8)

Higher education - master's degree |__| (9)

Higher education - doctoral degree |__| (10)

No matching |__| (11)

Prefer not to answer |__| (98)

Do not know| __| (99)

2. MINI MENTAL STATE EXAMINATION (MMSE)

Warning: cognitive impairment if: *illiterate ≤ 15, 1 to 11 years of schooling ≤ 22 and> 11 years of schooling ≤ 27*

- If the participant obtains a score indicative of cognitive impairment or is unable to sign the informed consent, obtain the information through his/hers legal guardian or the signature of two witnesses and his/hers identification (name and citizen's card).

2.1. Orientation *(1 point for each correct answer.)*

2.1.1. What year is it? |__| 2.1.2. What month is it? |__| 2.1.3. What day of the month is it? |__|

2.1.4. What day of the week is it? |__| 2.1.5. What season is it? |__|

2.1.6. What country are we in? |__| 2.1.7. In which district do you live? |__| 2.1.8. In what land do you live? |__|

2.1.9. What house are we in? |__| 2.1.10. What floor are we on? |__| **2.1.11. Score:** |__|

2.2. Retention *(1 point for each word correctly repeated.)*

"I will say three words. I want you to repeat them after me, but only after I say them all. Try to memorize them."

- Pear - Cat - Ball **Score:** |__|

2.3. Attention and calculation *(1 point for each correct answer. If the participant answers one question incorrectly but then continues to subtract correctly, the subsequent answers are considered correct. Stop at the end of 5 answers.)*

"Now I am going to ask you to tell me how much is 30 minus 3, and then to the number you find, you subtract 3 and repeat this until I tell you to stop."

27 |__| **24** |__| **21** |__| **18** |__| **15** |__| **Score:** |__|

2.4. Recall *(1 point for each correct answer.)*

"Try to say the three words I previously asked you to memorize."

- Pear - Cat - Ball **Score:** |__|

2.5. Language *(1 point for each correct answer.)*

"What is this called? Show the objects:

a. Clock |__| b. Pencil |__| **Score:** |__|

c. "Repeat the phrase that I am going to say: “No ifs, ands, or buts” **Score:** |__|

d. "When I give you this sheet of paper, take it in your right hand, fold it in half and put it on the table"; give the sheet holding with both hands.

- Holds it in the right hand |__| - Folds in half |__| - Puts it on the table |__| **Score:** |__|

e. "Read what is on this card and do what it says there". Show a card with the clear and legible phrase, "CLOSE YOUR EYES". If the participant is illiterate the phrase is read.

- Closed his/hers eyes **Score:** |__|

f. "Write a whole sentence here." The sentence must contain subject and verb and make sense. Grammatical errors do not affect the score.

Sentence:____ **Score:** |__|

2.6. Visual Construction *(1 point for each correct answer.)*

You must copy a drawing. Two partially overlapping pentagons; each one should have 5 sides, two of which intersect. Ignore tremor or rotation.

Copy:

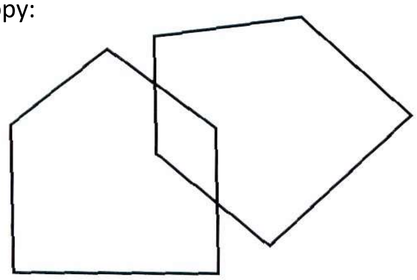

Score: |__|

2.7 Total score (maximum 30 points): |__|__|

<u>It is considered with cognitive impairment:</u> *no education ≤ 15, 1 to 11 years of education ≤ 22 and> 11 years of education ≤ 27*

- If the participant obtains a score indicative of cognitive impairment or is unable to sign the informed consent, obtain the information through his/her legal guardian or the signature of two witnesses and his/her identification (name and citizen's card).

3. WHO PROVIDES INFORMATION

Him/herself |__| (0) A family member |__| (1) Other person |__| (2)

4. CURRENTLY, WHAT IS YOUR PROFESSIONAL ACTIVITY?

Active|__| (0) If active go to question **4.1.**

Not active |__| (1) If not active go to question **4.2.**

4.1. You have a job.

4.1.1. If you have a job, what is your main occupation?
- Also choose this option if you work without being paid to a family member you live with.
- Indicate your profession as completely as possible or describe the main tasks you perform.
- If you have more than one profession, respond in relation to the one that currently occupies you the most time (greater number of hours).
Description of the profession:

____

____

4.1.2. Do you work full time?

Yes |__| (0) No |__| (1)

Prefer not to answer |__| (8)

4.2. *Choose this option only if you had a job and are now retired. If you have never worked, but receive a pension choose the option "**Another situation of inactivity**".*

Are you retired? |__| (0) or with early retirement? |__| (1) unemployed? |__| (2)

Another situation of inactivity |__| (3)

4.2.1. What was your main occupation?
- Indicate your profession as completely as possible or describe the main tasks you have performed. If you have had more than one profession, respond in relation to the one that has occupied you the most time (greater number of hours).
Description of the profession:

____

____

4.3. Other occupations

Does domestic chores |__| (0)

Does civic or community service (compulsory or voluntary) |__| (1)

Prefer not to answer |__| (8)

Do not know |__| (9)

5. LIFESTYLES

5.1 ACTIVITY / PHYSICAL EXERCISE

- We are interested in knowing the different types of physical activity that people do in their daily lives. The questions that I am going to ask you, refer to the immediately preceding week, considering the time during which you have been physically active. Please answer every question, even if you do not consider yourself a physically active person. We will ask you about the activities carried out in your professional activity and on commutes, activities related to housework and activities that you did in your free time for recreation or practice of physical exercise/sport.

In answering the following questions consider the following:
- Vigorous Physical Activities: *refer to activities that require intense physical exertion which cause heavy breathing.*
- Moderate Physical Activities *refer to activities that require moderate physical exertion which cause you to breathe a little harder than usual.*
- When answering questions consider only the physical activities you do for at least 10 minutes in a row.

5.1.1. Please tell me, in the last 7 days, on how many days did you walk for at least 10 minutes in a row?

Number |__|days

5.1.2. How much, in total time, did you spend on walking, on one of those days?

|__|__|Hours |__|__|minutes

5.1.3. Please tell me, on a normal day, how much time do you spend sitting? This can include time spent at a desk, visiting friends, reading, studying or watching television.

|__|__|Hours |__|__|minutes

5.1.4. Please tell me, in the last 7 days, on how many days did you do <u>vigorous physical activities</u>, such as lifting heavy objects, farming, digging, aerobics, swimming, playing football, cycling at a fast pace?

Number |__|days

5.1.5. When you engage in <u>vigorous physical activities</u>, how much time on average do you normally spend on those activities per day?

|__|__|Hours |__|__|minutes

5.1.6. Please tell me, in the last 7 days, on how many days did you do <u>moderate physical activities</u> such as carrying light objects, hunting, carpentry, gardening, cycling at a normal pace or even tennis? Please do not include "walking".

Number |__|days

5.1.7. When you engage in <u>moderate physical activities</u>, how much time on average do you normally spend on those activities per day?

|__|__|Hours |__|__|minutes

5.2 TOBACCO USE/SMOKING HABITS

Consider any type of tobacco, with the exception of electronic cigarettes

5.2.1. Do you currently smoke?

No|__| (0) Yes|__| (1) Prefer not to answer |__| (8)

5.2.2. How often do you smoke?

Daily|__|(0) Weekly |__|(1) Monthly or less frequently |__|(2)

5.2.3. *For those reporting smoking daily:*

On average how many cigarettes do you smoke a day (number cigarettes)? |__|__|__|

5.3. DO YOU CONSUME ALCOHOLIC BEVERAGES?

5.3.1. No |__| (0) Yes |__| (1) Prefer not to answer |__| (8)

5.3.2. How often do you drink?

Everyday |__| (0) Two to six times a week |__| (1) Once a week |__|(2) Less than once a week |__| (3)

5.3.3. On average, how many drinks do you consume on a day when you drink alcohol? |__|__|

Prefer not to answer |__| (88)

5.4. ADHERENCE TO THE MEDITERRANEAN DIET PATTERN *(PREDIMED)*

		Criteria for 1 Point	Score
1	Do you use olive oil as main culinary fat?	Yes	
2	How much olive oil do you consume in a given day (including oil used for frying, salads, out-of-house meals, etc.)?	≥ 4 tablespoons	
3	How many vegetable servings do you consume per day? (1 serving: 200 g [consider side dishes as half a serving])	≥ 2 servings per day (≥1 raw or as a salad)	
4	How many fruit units (including natural fruit juices) do you consume per day?	≥ 3 per day	
5	How many servings of red meat, hamburger or meat products (ham, sausage, etc.) do you consume per day? (1 serving: 100-150 g)	< 1 serving per day	
6	How many servings of butter, margarine, or cream do you consume per day? (1 serving: 12 g)	< 1 serving per day	
7	How many sweet or carbonated beverages do you drink per day?	< 1 per day	
8	How much wine do you drink per week?	≥ 7 glasses per day	
9	How many servings of legumes do you consume per week? (1 serving: 150 g)	≥ 3 per week	
10	How many servings of fish or shellfish do you consume per week? (1 serving: 100-150 g fish or 4-5 units or 200 g shellfish)	≥ 3 per week	
11	How many times a week do you consume commercial sweets or pastries (not homemade) such as cakes, cookies, biscuits or custard?	<3 times per week	
12	How many servings of nuts (including peanuts) do you consume per week? (1 serving 30 g)	≥ 3 per week	
13	How many servings of nuts (including peanuts) do you consume per week? (1 serving 30 g)	Yes	
14	How many times a week do you consume vegetables, pasta, rice or other dishes prepared with a stew (tomato, onion, leek or garlic and olive oil)?	≥ 2 times per week	
**Good adherence to the Mediterranean diet**		**Final score ≥ 10**	

Adapted from Martínez-González MA, García-Arellano A, Toledo E, Salas-Salvadó J, Buil-Cosiales P, et al. (2012) A 14-Item Mediterranean Diet Assessment Tool and Obesity Indexes among High-Risk Subjects: The PREDIMED Trial. PLOS ONE 7(8): e43134. https://doi.org/10.1371/journal.pone.0043134

6. HEALTH STATUS

6.1 SELF-PERCEPTION OF HEALTH STATUS

- The next question refers to the perception of your health.
- Answer the question by choosing the most appropriate answer. If you are not sure how to respond, please give the answer that seems most appropriate to you.

In general, how is your health?

Very good |__| (0) Bad |__| (3)
Good |__| (1) Very bad |__| (4)
Moderate |__| (2) Prefer not to answer |__| (5) Do not know |__| (9)

7. CHRONIC DISEASES

7.1. Do you have any chronic illness or long-term health problem?
- Answer "Yes" if the health problem lasts or can last for more than 6 months. Consider health problems that you keep under control by taking medication, seasonal health problems (eg. allergies) or health problems caused by injury, congenital conditions or birth defects.

No |__| (0)   Yes |__| (1)   Prefer not to answer |__| (8)

7.2. Please indicate whether you have suffered from the following diseases during the past 12 months:

	No (0)	Yes (1)	Prefer not to answer (8)	Do not know (9)
**7.2.1.** Asthma (including allergic asthma)				
**7.2.2.** Chronic bronchitis, chronic obstructive pulmonary disease or emphysema				
**7.2.3**. Myocardial infarction (or heart attack) or chronic consequences of a myocardial infarction				
**7.2.4.** Coronary heart disease or angina pectoris (*Consider all heart artery diseases.) Do not consider the chronic consequences of myocardial infarction.*				
**7.2.5.** High blood pressure, i.e., hypertension				
**7.2.6.** Stroke or chronic consequences of a stroke				
**7.2.7.** Osteoarthritis (or degenerative joint disease)				
**7.2.8.** Low back pain or other chronic back problems				
**7.2.9.** Neck pain or other chronic neck problems				
**7.2.10.** Diabetes				
**7.2.11.** Liver cirrhosis. Chronic liver disease. *Includes all types of cirrhosis (even non-alcoholic).*				
**7.2.12.** Allergies (rhinitis, hay fever, allergic conjunctivitis, dermatitis, food allergies or other allergies). *Do not consider allergic asthma.*				
**7.2.13.** Chronic kidney conditions, including kidney failure. *Kidney stones should only be considered if you think it is a chronic or prolonged problem.*				
**7.2.14.** Urinary incontinence or bladder control problems				
**7.2.15.** Depression				
**7.2.16.** Another? If "Yes", register the name	______________________________			

7.3. REGISTRATION OF MEDICINES AND NUTRITIONAL SUPPLEMENTS

What medications and nutritional supplements do you take daily? - *Register name and daily intake.*

Name of the medicine		No. of daily doses	Name of the nutritional supplement		No. of daily doses
**1**			**1**		
**2**			**2**		
**3**			**3**		
**4**			**4**		
**5**			**5**		
**6**			**6**		
**7**			**7**		
**8**			**8**		
**9**			**9**		
**10**			**10**		

8. NUTRITIONAL STATUS

8.1 ANTHROPOMETRY

8.1.1. Weight *- Record weight in light clothing and without shoes* |__|__|__| , |__|(kg)

8.1.2. Height *- Record height without shoes* |__|__|__| , |__| (cm)

- If you cannot measure, measure the: Hand length: |__|__| , |__| (cm)

8.1.3. Mid-upper arm circumference |__|__|__| , |__| (cm)

8.1.4. Waist circumference |__|__|__| , |__| (cm)

8.1.5. Calf circumference |__|__|__| , |__| (cm)

8.1.6. Triceps skinfold thickness |__|__| , |__| (mm)

8.2. FUNCTIONAL INDICATORS

8.2.1. Handgrip strength

1st measurement |__|__|__| , |__| (kgf)

2nd measurement |__|__|__| , |__| (kgf)

3rd measurement |__|__|__| , |__| (kgf)

8.2.2. Gait speed *(time required to walk 4.6m)* |__|__|__| , |__| (seconds)

8.2.3. ***Mini-nutritional assessment - Short Form***[a]

- Complete the screen by filling in the boxes with the appropriate numbers. Total the number to for the final screening score.

A. Has food intake declined over the past 3 months due to loss of appetite, digestive problems, chewing or swallowing difficulties? |__|

0 = severe decrease of food intake
1 = moderate decrease of food intake
2 = no decrease of food intake

B. Weight loss during the last 3 months? |__|

0 = weight loss greater than 3 kilos
1 = does not know
2 = weight loss between 1 and 3 kilos
3 = no weight loss

C. Mobility |__|

0 = bed or chair bound
1 = able to get out of bed/chair but does not go out
2 = goes out

D. Has suffered psychological stress or acute disease in the past 3 months? |__|

0 = yes
2 = no

E. Neuropsychological problems |__|

0 = severe dementia or depression
1 = mild dementia
2 = no psychological problems

F1. Body Mass Index (BMI = weight [kg] / height^2 [m^2]) |__|

0 = BMI < 19
1 = 19 ≤ BMI < 21
2 = 21 ≤ BMI < 23
3 = BMI ≥ 23

[a]Adapted from: Vellas B *et al. Overview of the MNA® - Its History and Challenges*. J Nutr Health Aging 2006;10:456-465. Rubenstein LZ *et al. Screening for Undernutrition in Geriatric Practice: Developing the Short-Form Mini Nutritional Assessment (MNA-SF)*. J. Geront 2001;56A: M366-377.)

IF BMI IS NOT AVAILABLE, REPLACE QUESTION F1 WITH QUESTION F2.
DO NOT ANSWER QUESTION F2 IF QUESTION F1 IS ALREADY COMPLETED

F2. Calf circumference (CC) in cm |__|
0 = CC less than 31
3 = CC 31 or greater

Screening score (max: 14 points) |__|__|

- 12-14 points: normal nutritional status
- 8-11 points: at risk of malnutrition
- 0-7 points: malnourished

8.2.4. FRAGILITY – FRIED SCALE

8.2.4.1. WEIGHT LOSS

>4,5 kg in the last year, unintentionally No |__| (0) Yes |__| (1) Do not know |__| (9)

8.2.5. EXHAUSTION

8.2.5.1. "All I do requires a lot of effort." Last week, how many times have you felt like this? |__|__|

8.2.5.2. "I cannot go on"

Never or almost never (<1 day) |__| (0)
Sometimes (1 – 2 days) |__| (1)
Sometimes (3 – 4 days) |__| (2)
Almost always or always |__| (3)

9. COABITATION

- Please tell me who you live with:

Degree of kinship	Sex	Age	Degree of kinship	Sex	Age

10. SKIN PHENOTYPE

What is your skin type?

a. Light skin, blue eyes, freckles (always burns and never tans) |__| (0)
b. Light skin, blue eyes, green or light brown, blond or red hair (always burns and sometimes tans) |__| (1)
c. As the average of the normal white skin-types (sometimes a mild burn, tans gradually and evenly |__| (2)
d. Clear or light brown skin, dark brown hair and dark eyes (rarely burns, tans moderately) |__| (3)
e. Brown skin (very rarely burns, tans a lot) |__| (4)
f. Dark skin (never burns, tans very easily, deeply pigmented). |__| (5)

11. Finally, I ask you to indicate the approximate value of your household's total monthly income (considering all sources of income) net after deducting all taxes.

|__|__|__||__|__|__|€ Prefer not to answer |__| (8)

INFORMATION TO THE PARTICIPANT

The project ***"Nutritional Strategies Facing an Older Demographic: The Nutrition UP 65 Study"*** is being conducted by the Faculty of Nutrition and Food Sciences of the University of Porto.

This study aims to improve knowledge regarding older Portuguese adults' nutritional status, specifically about malnutrition, obesity, sarcopenia, fragility, vitamin D status, hydration and urinary sodium and potassium excretion. The main benefit of participating in this study is the possibility of finding out the test results regarding your nutritional status. Yours and other participants test results and will also allow the development of food and nutrition education programs adapted to this age-group.

We will ask you to answer questions whose content is important to the study. All procedures are simple and will be performed by professionals previously trained for this purpose.

Potential risks and discomforts relating to your participation are not anticipated, other than those associated with blood and urine sample collection, bodyweight, height, triceps skinfold, arm and leg circumferences measurements as well as with handgrip strength and gait speed assessment. There will be a questionnaire that will last about 20 minutes.

All of the information you provide to us is confidential and its privacy will always be guaranteed.

We inform that **your participation in this study is voluntary and that you have the right to refuse to participate or to withdraw, without penalty or consequences of any kind.**

Thank you in advance for your attention.

Principal Investigator Contact:

Xxxxxxxxx (Daily Spin) Telephone: xxxxxxx

FCNAUP, Telephone: 225074320

DECLARATION OF CONSENT

Considering the latest version of the "Declaration of Helsinki" of the World Medical Association

The Nutrition UP 65 Study|Nutritional Strategies Facing an Older Demographic

I, the undersigned,
____,

declare that I have understood the explanation given to me concerning the study that is intended to be carried out. I was given the opportunity to ask the questions that I thought necessary, and I got a satisfactory answer to all of them.

I have been informed that, according to the recommendations of the Helsinki Declaration, the information or explanation given to me has addressed the objectives, methods, expected benefits, potential risks and possible discomfort. In addition, I have been told that I have the right to refuse my participation in the study at any time, without penalty or consequences of any kind.

Therefore, I consent to the application of the procedures proposed by the investigator.

Date: _____ / _________________ / 201___

Volunteer Signature:

____

Signature of the investigator in charge:

____
